# Effect of protein oxidation on the quality of abalone (*Haliotis discus hannai*) during frozen storage under different packaging conditions

**DOI:** 10.1016/j.fochx.2025.102357

**Published:** 2025-03-10

**Authors:** Siyuan Zhu, Chen Zhang, Yijun Liu, Dan Jiang, Qiancheng Zhao, Xiqin Mao, Xia Hu, Bohai Jiang

**Affiliations:** a College of Food Science and Engineering, Dalian Ocean University, 52 Heishi Jiao Street, Dalian 116023, China; b Dalian Center for Certification and Food and Drug Control, No.888A Huanghe Road, Shahekou District, Dalian 116021, China; c Dalian Product Quality Inspection and Testing Institute Co., Ltd., No.68-2 Wansui Road Shahekou District, Dalian 116021, China

**Keywords:** Abalone, Protein oxidation, Quality regulation, Correlation, Packaging

## Abstract

This study investigated the effects of protein oxidation under vacuum packaging, ice-coating, air-permeable polyvinyl chloride and non-packaging on sensory quality of abalone during 24 weeks at −20 °C. During storage, carbonyl content of protein increased (1.64 to 3.12–4.13 nmol/mg), sulfhydryl content decreased (20.48–29.94 %), surface hydrophobicity increased (19.50–40.24 %) and Ca^2+^-ATPase activity decreased (21.13–54.93 %). Protein secondary structures was converted into random coils, UV absorption of chromogenic groups reduced indicated tertiary structure and functional degradation. Compared to others, vacuum packaging decreased carbonyl content (3.41–24.46 %) and slowed down the oxidation process among 9 ∼ 19w, while ice-coating increased L* value (4.12–12.75 %) and maintained freshness among 8 ∼ 19w. The L* value (*r* = −0.89) and centrifugal loss (*r* = 0.95) were significantly correlated with carbonyl content, and hardness (*r* = −0.70) was significantly correlated with surface hydrophobicity (*p* < 0.01). Thus, 0 ∼ 8w is the effective period in protein degradation regulation considering oxidation indicators and quality control by WHC trend.

## Highlights

• The color quality of abalone is influenced by protein carbonyl modifications.
• The texture of abalone is affected by the hydrophobicity of its protein.
• The impact of oxidative regulation on abalone protein should not exceed 8 weeks.
• Vacuum packaging abalone is more effective in controlling protein oxidation process.
• Ice-coating is effective for preserving the color and texture quality of abalone.

## 1 Introduction

Abalone (*Haliotis discus hannai*) is a precious univalve shellfish, ranking first in the list of "four treasures of seafood" (Chan et al., 2012). According to the statistical aquaculture and fisheries yearbooks, the global abalone aquaculture industry has developed rapidly, and the output value has increased from $ 4 million at the beginning of the 20th century to more than $ 1.8 billion in recent years (Hernández-Casas et al., 2023). As the world's largest abalone exporter and consumer, China's abalone aquaculture production accounts for nearly 90 % of the world's total production (Gao, Liu, et al., 2023). Freezing can inhibit microbial reproduction and prolong the shelf life of abalone, which is widely used in abalone preservation. However, with the prolongation of frozen storage, abalone is prone to quality deterioration such as color change, texture decline and water loss, the edible value is greatly reduced, causing huge economic losses to the abalone industry. Therefore, it is particularly important to deeply explore the mechanism of quality change in frozen abalone and maintain the freshness of abalone.

It has been reported that microbial growth, lipid and protein peroxidation are the main factors leading to the quality deterioration of aquatic products during processing and storage. As the main component of muscle tissue of aquatic products, protein is the material basis that determines the nutritional value, sensory quality and flavor characteristics of aquatic products (Yu et al., 2024). Moderate oxidation can promote the unfolding of protein structure and enhance the gelation and emulsification of meat, excessive oxidation will destroy the structure and functional properties of proteins, which will lead to the loss of freshness and nutritional value of meat, and even produce toxic secondary oxidation products (Günal-Köroğlu et al., 2024; Xiong & Guo, 2020). Therefore, controlling protein oxidation is the key to the quality regulation of abalone.

Reactive oxygen species (ROS) are the primary oxidative factors inducing covalent modifications in proteins (Nawaz et al., 2022). The oxidation process involves carbonylation, S-nitrosylation, and aromatic hydroxylation, with the resulting products serving as key indicators for assessing the degree of protein oxidation (Zhang et al., 2024). Packaging strategies can regulate protein‑oxygen contact, reduce electron transfer in chromogenic groups and inhibit photo-oxidation. In order to control protein oxidation, Lai et al. (2023) reported that HiO_2_ modified atmosphere packaging (MAP) can achieve color protection on beef steaks. Delles and Xiong (2014) reported that High oxygen atmosphere promotes the oxidation of meat protein, increases hydration but decreases water binding. Abalone is high-value shellfish with rich protein and low lipid, the mechanisms underlying protein oxidation during storage, the direct impact of oxidation on abalone quality and the extent to which packaging can regulate oxidation remain unclear. It is of commercial significance to clarify the oxidation process and make quality regulation.

Building on these findings, we investigated the effects of different packaging methods—vacuum, ice-coating, air-permeable polyvinyl chloride (PVC), and non-packaging—on the oxidation and structural changes of abalone during frozen storage. Specifically, protein oxidation was assessed through sodium dodecyl sulfate-polyacrylamide gel electrophoresis (SDS-PAGE), carbonyl content (2,4-dinitrophenylhydrazine, DNPH assay), sulfhydryl content (Ellman's method), surface hydrophobicity (bromophenol blue, BPB binding), and Ca^2+^-ATPase activity, with detection limits of 0.1 nmol/mg for carbonyl content and 0.01 U/mg for Ca^2+^-ATPase activity. Structural changes of proteins were analyzed using Fourier Transform Infrared Spectroscopy (FT-IR) and ultraviolet (UV) spectrophotometry. The correlation between quality attributes, including color, texture and water holding capacity (WHC), and oxidation indicators was evaluated using Pearson correlation coefficients. This study reveals the direct effects of protein oxidation on the color, texture and WHC quality of abalone during frozen storage, while quantifying the effectiveness of oxygen-controlled packaging in regulating oxidative processes. The results offer a theoretical foundation for optimizing packaging strategies to maintain freshness and extend the shelf life of frozen abalone.

## 2 Materials and methods

### 2.1 Materials and sample preparation

Fresh abalone, in the rapid growing period, purchased from the local aquatic products wholesale market in Dalian, Liaoning Province, China, with an average weight of 60.86 g and a length of 71.11 mm. After purchase, it is stored in a seawater tank and transported to the in vivo laboratory within 1 h.

### 2.2 Packaging

Fresh abalones were washed and drained after shell and viscera were removed. Five abalones were randomly selected and allocated to vacuum packaging (VP), ice-coating packaging (IP), air-permeable polyvinyl chloride packaging (AP) and non-packaging (NP) systems. For VP, the abalone was sub-packed into a flexible polyvinylidene chloride type vacuum bag and vacuum sealed at 1 bar pressure to control the vacuum degree to more than 90 %. For IP, abalone was frozen at −20 °C for 12 h, and then immersed in pure water for 30s, so that pure water quickly condensed into ice on the surface of abalone, and the amount of ice plating was controlled to be 15 ± 2 %. For AP, the abalone was sealed in air-permeable polyvinylchloride bag. For NP, the abalone does not need packaging. All abalones in each group were placed without stacking and frozen at −20 ± 2 °C in a plate freezer to ensure consistent freezing rates and minimize temperature fluctuations. The freezing rate of abalones was monitored about 1 °C/min. Thawed the abalones in 4 °C at 1, 2, 3, 4, 6, 8, 12, 16, and 24 weeks for the determination of various quality indicators, including protein oxidation, structural changes, and sensory attributes.

### 2.3 SDS-PAGE

SDS-PAGE analysis was performed using 10 % separation gel and 5 % concentrated gel. The protein solution was adjusted to 3 mg/mL and mixed with the loading buffer 1:1, water bath heating for 10 min, and compared with the pre-stained protein Marker (25–300 kDa). The injection volume was 10 μL, and the electrophoresis condition was 120 V. After electrophoresis, Coomassie Brilliant Blue G-250 (0.1 %) staining was used to dye the gel for 30 min and then decolored to clear bands.

### 2.4 Extraction of myofibrillar proteins (MPs)

MPs were extracted from abalone muscles according to the method of Zhang et al. (2016) with minor modification. Accurately weighed 3 g of abalone minced muscles was mixed with 15 mL of cold sodium phosphate buffer (10 mM, 50 mmol/L Na_2_HPO_4_, 50 mmol/L NaH_2_PO_4_, pH 7.5), homogenized and centrifuged (10,000 r/min, 10 min, 4 °C), the supernatant was discarded and this step was repeated three times. Afterward, the precipitate was dissolved in cold sodium phosphate buffer containing NaCl (0.6 M, pH 7.0) homogenized and centrifuged (10,000 r/min, 10 min, 4 °C). this step was repeated twice. The combined supernatant was MPs. The content of MPs was determined by bicinchoninic acid (BCA) method.

### 2.5 Determination of oxidative indicators

#### 2.5.1 Determination of carbonyl content

The determination of carbonyl content according to the method of Aydin and Gokoglu (2014) with minor modification. 1 mL MPs solution (2 mg/mL) was mixed with 1 mL DNPH solution (10 mM), incubated in the dark for 1 h. Afterward, 1 mL trichloroacetic acid (TCA, 20 %, w/w) was added to the mixture, precipitation and centrifugation (10,000 r/min, 10 min, 4 °C), then washed with ethanol: ethyl acetate solution (1:1, v/v) three times. The pellets were dissolved in 6 M guanidine hydrochloride. The supernatant absorbance was measured at 370 nm.

#### 2.5.2 Determination of sulfhydryl content

The determination of sulfhydryl content according to Ellman's method. 0.5 mL MPs solution (4 mg/mL) was mixed with 4.5 mL phosphate buffer (0.1 M, 8 mol/L urea, 0.6 mol/L KCl, 10 mmol/L ethylenediaminetetraacetic acid (EDTA, pH 7.5). Afterward, 4 mL 5,5’-Dithion-bis-2-nitrbenzoic acid (DTNB, 20 %, w/w) was added to the mixture. The absorbance was measured at 412 nm after incubating for 30 min in water bath at 40 °C.

#### 2.5.3 Determination of surface hydrophobicity

The determination of surface hydrophobicity content is as follows: 1 mL MPs solution (4 mg/mL) was mixed with 200 μL BPB (1 mg/mL), The blank group was replaced with phosphate buffer (20 mM, pH 6.0). The mixture was shaken for 30 mins and centrifuged (5000 r/min, 10 min, 4 °C), and the supernatant absorbance was measured at 595 nm. The surface hydrophobicity was expressed by the content of BPB combined.

#### 2.5.4 Determination of Ca^2+^-ATPase activity

The Ca^2+^-ATPase activity was determined by the Ca^2+^-ATPase assay kit (A070–4-1, Nanjing Jiancheng Bioengineering Institute, Jiangsu, China).

### 2.6 Determination of MPs structure

#### 2.6.1 Determination of secondary structure

MPs were scanned by Fourier transform infrared spectrometer (370DTGS, Agilent, USA). Test parameters are as follows: spectrum scanning range 500–4000 cm^−1^, resolution 4 cm^−1^, SNR 50000:1, scanning 64 times. The percentage contents of α-helix, β-sheet, β-turn, and random coil were calculated by Gaussian fitting of Peakfit 4.0 software.

#### 2.6.2 Determination of tertiary structure

1 mg/mL MPs solution was measured in an ultraviolet spectrophotometer (UV-1200, Shanghai Mapada Instrument Co., Ltd., Shanghai, China). The test parameters were as follows: scanning wavelength range of 220–380 nm, scanning rate of 2 nm/min.

### 2.7 Determination of sensory characteristics

#### 2.7.1 Determination of color

Abalone muscle was measured by portable colorimeter (SC-10, Sanen Technology Co., Ltd., Shenzhen, China), and the average values of L* (brightness), a* (redness/greenness) and b* (yellowness/blueness) were recorded.

#### 2.7.2 Determination of texture

Abalone muscle tissue was cut into 1 cm × 1 cm × 1 cm cuboid, and the hardness, elasticity and chewability of abalone muscle were measured by texture instrument (TA-300 W, Saicheng Electronic Technology Co., Ltd., Shandong, China). The parameters were set as follows: P/0.5 probe, test speed 60 mm/min, shape variable 30 %, trigger force 1.5 N, and the probe was measured twice at an interval of 1 s.

#### 2.7.3 Determination of WHC

Thawing loss: The quality of abalone muscle before thawing (m_1_) and after thawing (m_2_) was weighed. Thawing loss (%) = (m_1_ - m_2_) / m_1_ × 100.

Centrifugal loss: The abalone muscle was cut into small pieces of 2 cm × 2 cm × 2 cm, wrapped in filter paper and centrifuged (5000 r/min, 10 min, 4 °C), and the quality before centrifugation (m_1_) and after centrifugation (m_2_) was weighed. Centrifugal loss (%) = (m_1_- m_2_) / m_1_ × 100.

Boiling loss: The abalone muscle was cut into small pieces of 2 cm × 2 cm × 2 cm, boiled in water bath at 100 °C for 10 min, cooled down to room temperature and dried with filter paper, and the quality before boiling (m_1_) and after boiling (m_2_) was weighed. Boiling loss (%) = (m_1_- m_2_) / m_1_ × 100.

#### 2.7.4 Microstructure observation

The abalone muscles connected to the shell column were longitudinally cut into 0.5 cm × 0.5 cm × 0.3 cm cuboid, wrapped with paraffin, sliced and dewaxed. The slices were stained with hematoxylin and eosin (H&E) successively, then dehydrated and sealed, and the muscle fiber structure was observed by inverted fluorescent microscope (DMI 3000B, Leica Microscope Co., Ltd., Shanghai, China).

### 2.8 Statistical analyses

All experiments were repeated three times in parallel, and the data were expressed as mean ± standard deviation. SPSS Statistics 21.0 was used for univariate ANOVA analysis, and *p <* 0.05 indicated significant difference. Correlation analysis was performed using the Pearson coefficient two-tail test. Chart using Origin 2021 software.

## 3 Results and discussion

### 3.1 SDS-PAGE

**Fig. 1:**
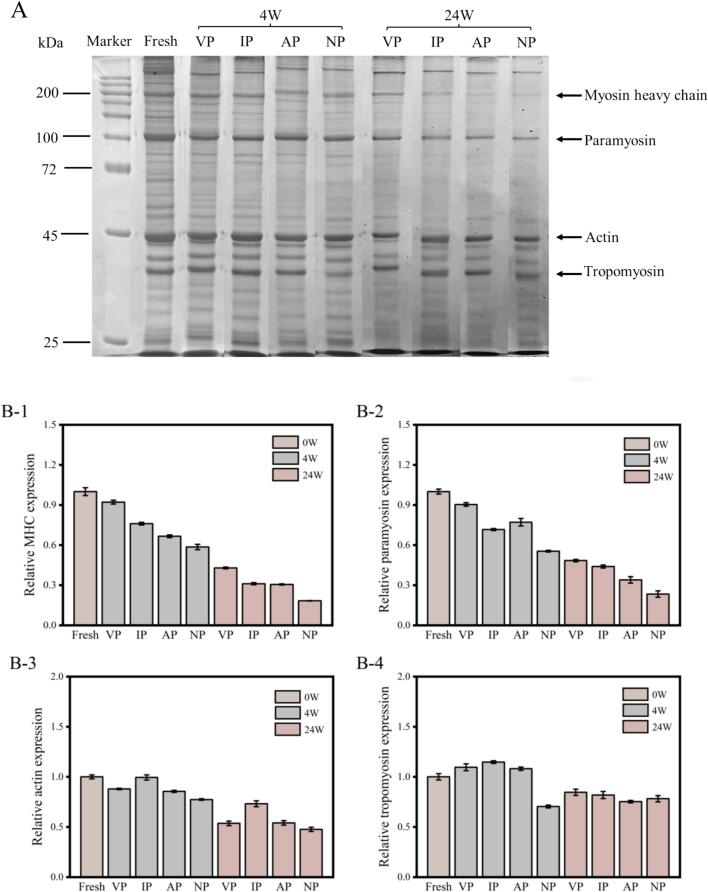
SDS-PAGE electrophoretic protein profiles (A) and relative protein expression levels (B) of abalone at 0, 4, and 24 weeks during −20 °C storage under different packaging. Note: B-1: Myosin heavy chain (MHC); B-2: Paramyosin; B-3: Actin; B-4: Tropomyosin. VP: vacuum packaging, IP: ice-coating packaging, AP: air-permeable polyvinyl chloride packaging, NP: non-packaging.

The electrophoresis pattern of abalone muscle proteins (25–300 kDa) with different packaging under frozen storage is shown in Fig. 1A. Major protein bands included myosin heavy chain (MHC, 200 kDa), paramyosin (100 kDa), actin (45 kDa), and tropomyosin (35 kDa) (Li et al., 2025). Semi-quantitative analysis in Fig. 1B revealed a significant decrease (*p* < 0.05) in the intensity of four main protein bands of all groups during storage, while bands at 25–35 kDa intensified, indicating the degradation of high-molecular-weight proteins into smaller peptides, which probably due to catheptic enzyme activity and oxidative fragmentation. The VP group exhibited stronger MHC and paramyosin band intensities compared with other packaging. It is attributed VP can reduce oxygen exposure, thereby reducing ROS production and oxidative damage (Tao et al., 2007). In addition, anaerobic conditions inhibited oxidase enzyme activity, slowed protein fragmentation (Santos et al., 2009). This protective effect preserved the structural integrity and functionality of MHC and paramyosin.

### 3.2 Changes in oxidative indicators

#### 3.2.1 Changes in the contents of carbonyl

**Fig. 2:**
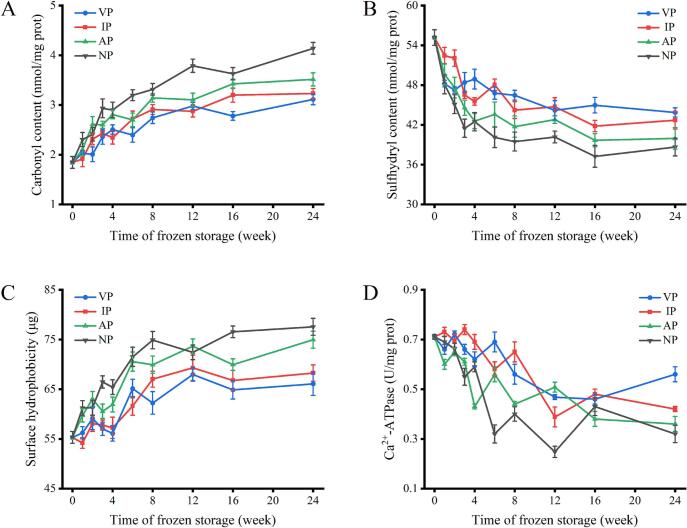
Changes in carbonyl content (A), sulfhydryl content (B), surface hydrophobicity (C) and Ca^2+^-ATPase activity (D) of abalone protein during −20 °C storage under different packaging.

The amino groups on protein side chains are susceptible to ROS attack, converting to carbonyl groups during freezing, so carbonyl content is a key indicator of protein oxidation (Zhang et al., 2022). As shown in Fig. 2A, the carbonyl content of fresh abalone was 1.64 nmol/mg. During storage, carbonyl content increased significantly (*p* < 0.05) in four groups due to freeze-concentration effects and ice crystal-induced cell disruption, which elevated oxidant concentrations and accelerated protein oxidation (Wu et al., 2024). At 24 weeks, carbonyl content in VP, IP, and AP groups was 3.12, 3.23, and 3.51 nmol/mg, significantly lower than NP (4.13 nmol/mg, *p* < 0.05). Compared with other packaging, the VP group relative decreased carbonyl content of 3.41–24.46 %. Comparisons conducted before the microbial index reached the rejection point (data not shown) revealed that the carbonyl content of the VP group at 24 weeks was equivalent to that of the IP group at 15 weeks, indicating that the VP group delayed the oxidation process by 9 weeks compared to the IP group. Similarly, the oxidation process was delayed by 19 weeks compared to the NP group. These results indicate that proteins are more prone to carbonylation in high‑oxygen environments, while packaging effectively regulates free radical oxidation rates, thereby controlling carbonylation reactions. This result is consistent with previous findings that high‑oxygen storage increases carbonyl content compared to vacuum packaging (Li et al., 2022).

#### 3.2.2 Changes in the contents of sulfhydryl

The sulfhydryl group is the main functional group of MPs and commonly involved in protein oxidation and conformational stability (Du, Cao, et al., 2022). The sulfhydryl content of fresh abalone was 55.17 nmol/mg in Fig. 2B. The sulfhydryl content of abalone in the four groups decreased rapidly during 0 ∼ 8 weeks of frozen storage, and decreased slowly during 8 ∼ 24 weeks. At 8 weeks, the sulfhydryl content had lost 77.08–95.04 % of the total decrease in entire freezing period. According to the stability of sulfhydryl content, it is speculated that 8 weeks is a key transition stage in protein degradation. The initial rapid decline may be driven by freeze-concentration effects and ice crystal growth, which disrupted cellular integrity and accelerated disulfide bond formation. The subsequent slowdown may result from protein aggregation and depletion of reactive sites, limiting further covalent modifications (Benjakul et al., 2003). At 24 weeks, sulfhydryl content in VP, IP, and AP decreased by an average of 20.48 %, 22.60 %, and 27.46 %, respectively, significantly less than NP (29.94 %, *p* < 0.05). Thus, the packaging of VP, IP, and AP effectively mitigate protein conformational changes and sulfhydryl exposure. Among them, VP demonstrates superior efficiency in inhibiting sulfhydryl oxidation.

#### 3.2.3 Changes in the surface hydrophobicity

Hydrophobic residues are usually buried in the protein structure. When the protein is unfolded or denatured, the surface hydrophobicity will increase (Lan et al., 2021). In fresh abalone, the surface hydrophobicity was measured at 55.29 μg/mg in Fig. 2C, indicating the baseline level of protein structure integrity. At 24 weeks, the surface hydrophobicity of VP, IP, AP and NP increased by an average of 19.50 %, 23.42 %, 35.56 % and 40.24 %, respectively. The changes in hydrophobicity reflect protein folding, unfolding and polymerization under external (Liu et al., 2024). Compared with other packaging, the VP group relative decreased the surface hydrophobicity among 3.17–14.79 %, demonstrating superior protein preservation. It is speculated that VP can reduce oxygen exposure, inhibiting protein cross-linking, unfolding and hydrophobic residue exposure. In contrast, other packaging methods permit oxygen penetration, promoting ROS formation, inducing disulfide bond formation and covalent cross-linking, leading to protein unfolding and hydrophobic residue exposure. This result consistent with previous findings on protein structural changes in high‑oxygen environments (Chen et al., 2024).

#### 3.2.4 Changes in the Ca^2+^-ATPase activity

Ca^2+^-ATPase activity is an important index to measure the integrity and degeneration of myosin. The Ca^2+^-ATPase activity of fresh abalone was 0.71 U/mg in Fig. 2D. During frozen storage, the Ca^2+^-ATPase activity content in four groups showed downward trend, due to the changes in myosin structure induced by ice crystal growth and ion concentration increase, the changes in tertiary structure induced by protein interaction, and the exposure of hydrophobic groups between proteins (Lin et al., 2019). At 24 weeks, Ca^2+^-ATPase activity of VP, IP, AP and NP decreased by an average of 21.13 %, 40.85 %, 49.30 % and 54.93 %, respectively, demonstrating packaging's protective effect. Among them, the VP group showed the smallest decline, attributed to minimized oxygen exposure, reduced ROS generation, and oxidative process, thereby limiting protein cross-linking, unfolding, and hydrophobic group exposure. Additionally, VP mitigated ice crystal formation and ion concentration changes, preserving myosin structural integrity and Ca^2+^-ATPase activity more effectively than other packaging.

### 3.3 Changes in MPs structure

#### 3.3.1 Change in protein secondary structure

**Fig. 3:**
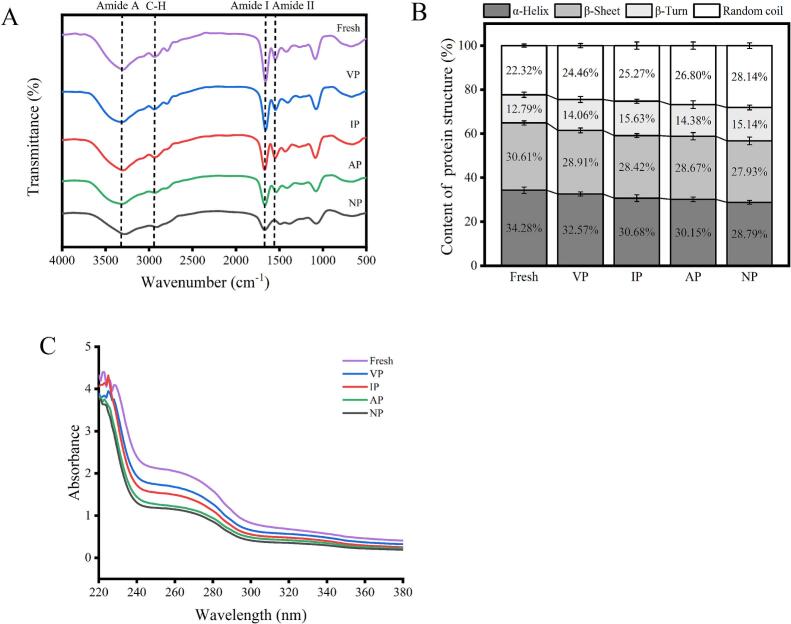
Changes in Fourier Transform Infrared Spectroscopy (FT-IR) spectra (A), content of secondary structure of myofibrillar proteins (B), and ultraviolet (UV) absorption spectrum of myofibrillar proteins (C) of abalone protein at 24 weeks during −20 °C storage under different packaging.

The FT-IR of abalone muscle protein at 24 weeks of frozen storage is shown in Fig. 3A. The infrared characteristic peaks of abalone include amide II band at 1500–1600 cm^−1^, amide I band at 1600–1700 cm^−1^, characteristic absorption peak at 2930 cm^−1^ and single strong peak at 3300 cm^−1^. There is a correlation between the amide I band and the protein skeleton conformation, which is often used to determine the relative content of the protein secondary structure, the results are shown in Fig. 3B. α-helix and β-sheet are the main secondary structures in abalone muscle proteins, which maintain the ordered and stable structure of MPs by hydrogen bonds. The content of α-helix and β-sheet in abalone MPs decreased, and the content of random coil and β-turn increased after frozen storage for 24 weeks. This may be due to the fact that the hydrogen bond was broken by water migration, ice crystal recrystallization and protein mechanical damage (Zhang et al., 2017), and the myofibrillar protein changed from an ordered structure to a disordered structure. Among them, the content of α-helix and β-sheet in the VP was higher than other packaging, indicating that VP effectively stabilized the protein secondary structure.

#### 3.3.2 Change in protein tertiary structure

Aromatic amino acids, including tyrosine (Tyr), tryptophan (Trp), and phenylalanine, exhibit UV absorption, with spectral changes reflecting protein conformational alterations (Wang et al., 2020). At 24 weeks, the UV spectrum of abalone muscle in Fig. 3C displayed a maximum absorption peak near 275 nm, corresponding to Tyr and Trp residues. Compared to fresh abalone, all groups exhibited a red shift, indicating microenvironmental changes around these residues due to protein unfolding, which altered polarity and hydrogen bonding, exposing them to a more hydrophilic environment (Lu et al., 2025). Additionally, the decreased UV intensity suggested protein aggregation and hydrophobic interactions during storage, creating steric hindrance and burying Trp residues, thereby reducing UV absorption (Lv et al., 2021). VP group showed UV absorbance closest to fresh abalone, indicating better preservation of the tertiary structure. This is attributed to VP minimizing oxygen exposure, reducing oxidative stress, and maintaining the native microenvironment around aromatic residues, thereby stabilizing protein conformation and UV absorption properties.

### 3.4 Determination of sensory characteristics

#### 3.4.1 Change in color

**Fig. 4:**
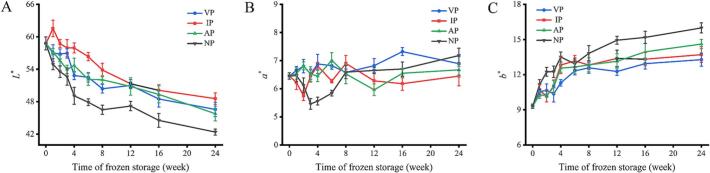
Changes in brightness *L*^⁎^(A), redness *a*^⁎^(B) and yellowness *b*^⁎^(C) of abalone during −20 °C storage under different packaging.

Color characteristic is an important sensory index of aquatic products, which is closely related to the chemical and oxidative state of myoglobin, the mechanical damage caused by ice crystals during freezing can promote the contact between ROS and myoglobin, accelerate the oxidation process and the color change of aquatic products (Bao et al., 2021). The changes in muscle characteristics of frozen abalone are shown in Fig. S1. The color of abalone muscle in four groups gradually darkened and turned yellow during the frozen storage, and the sensory quality decreased seriously. The L*, a* and b* value of frozen abalone was determined by colorimeter in Fig. 4. It is showed that with the prolongation of storage, the L* value of the four groups of abalone decreased significantly, the a* value not significantly change, and the b* value increased significantly (*p* < 0.05). At 24 weeks, the IP group relative increased the L* value among 4.12–12.75 % and slowed down color changes among 8 ∼ 19 weeks compared to other packaging. This is attributed to the ice-coating covered the surface of abalone. On the one hand, it inhibited the oxidation of myoglobin, and on the other hand, it increased the wettability of the body surface and increased the reflectivity of light.

#### 3.4.2 Change in texture

**Fig. 5:**
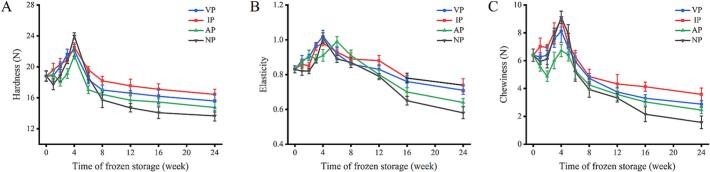
Changes in hardness (A), elasticity (B) and chewiness (C) of abalone during −20 °C storage under different packaging.

Texture characteristics are crucial for assessing aquatic product quality, as protein oxidation, cross-linking, and net charge changes directly affect texture (Domínguez et al., 2021). As shown in Fig. 5, the hardness, elasticity, and chewiness of abalone muscle in four groups initially increased, peaking at the 4th week, likely due to disulfide bond formation and covalent cross-linking, creating a dense gel network and hardening texture (Du, Wang, et al., 2022). Subsequently, texture declined as proteolytic enzymes degraded proteins. At 24 weeks, the IP group relative increased the hardness among 5.17–16.91 % and preserved the texture among 12 ∼ 16 weeks compared to other packaging. The superior performance of IP can be explained by its physical barrier effect. The ice layer minimizes direct contact between the abalone and freezing environment, thereby effectively slowing the formation of large ice crystals and mitigating mechanical damage to abalone muscles. Notably, it is speculated that texture loss in frozen abalone is influenced not only by protein oxidation but also by factors such as ice crystal formation, solute concentration, and exudate loss, this is consistent with previous research (Gao, Zhang, et al., 2023).

#### 3.4.3 Change in WHC

**Fig. 6:**
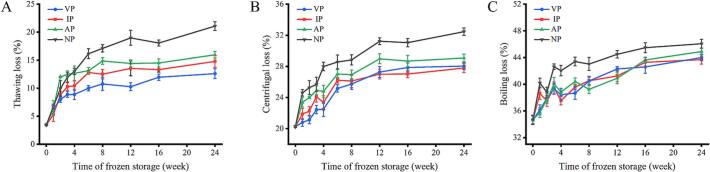
Changes in thawing loss (A), centrifugal loss (B), boiling loss (C) of abalone during −20 °C storage under different packaging.

WHC is closely related to cell structural integrity and protein structure, protein oxidation usually leads to a decrease in muscle WHC (Warner, 2023). In this study, abalone muscle WHC was assessed through thawing, centrifugal, and cooking losses, which are shown in Fig. 6. All groups showed significant increases in these losses during frozen storage (*p* < 0.05). At 8 weeks, the centrifugal loss of all groups had reached 70.25–77.82 % of the total loss within 24 weeks. Research indicates that ice crystals and internal stress can rupture cell membranes and enlarge intercellular spaces, causing fluid leakage, while oxidation disrupts hydrogen bonds, electrostatic interactions, and capillary forces, impairing water reorganization (Li et al., 2024). Among them, the NP group exhibited the lowest WHC, followed by AP, IP, and VP. It is speculated that the temperature differences and inevitable internal airflow during the flat plate freezing process promote the formation of large ice crystals in NP abalone. These large ice crystals increase solute ion concentration, accelerate protein denaturation, and reduce hydration surfaces. In contrast, the VP, IP, and AP groups inhibit ice crystal growth and water migration, thereby preserving the structure and function of proteins and maintaining higher WHC.

#### 3.4.4 Microstructure of muscle tissue

**Fig. 7:**
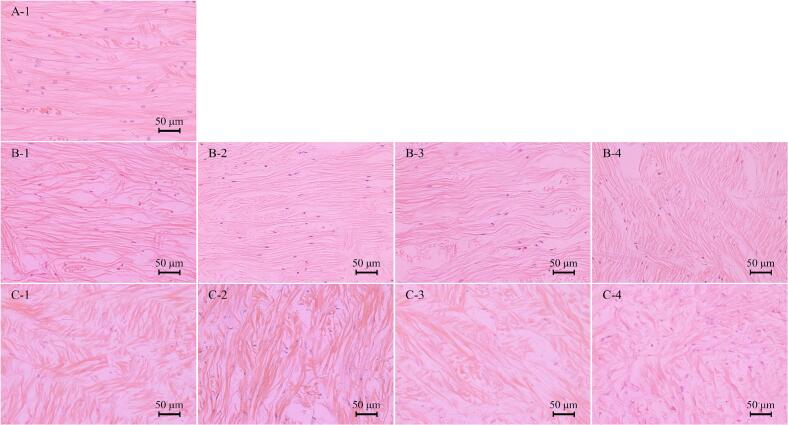
Longitudinal section in abalone muscle tissue at 0, 4, and 24 weeks during −20 °C storage under different packaging methods. (Note: A-1: Fresh abalone; B-1 to B-4: Abalone at the 4th week during −20 °C storage under VP, IP, AP, and NP; C-1 to C-4: Abalone at the 24th week during −20 °C storage under VP, IP, AP, and NP.)

The effect of protein oxidation on the muscle fiber structure of abalone can be observed through the microstructure of the tissue. According to the 3.4.2, the turning point of abalone quality change was 4 weeks. Therefore, the tissue longitudinal sections of frozen abalone muscle at 0, 4 and 24 weeks were observed in this study in Fig. 7. The muscle fibers of abalone were stained red, and the nucleus was stained blue-purple after H&E staining. The muscle fiber structure of fresh abalone is complete, and the muscle tissue is arranged neatly. With prolonged frozen storage, the abalone muscle fibers of four packages showed different degrees of deformation. In the 4th week, the muscle fibers in four groups gradually became irregular and gaps appeared, and the NP abalone muscle fibers even broke. At the 24th week, the muscle fiber structure of NP abalone appeared disordered and blurred. In contrast, packaging effectively mitigated damage to the muscle fibers. However, VP experienced slight muscle fiber rupture and deformation due to mechanical compression. Notably, the IP group maintained a relatively intact muscle fiber structure, as the ice layer not only avoided mechanical compression but also minimized ice crystal-induced damage during storage. This structural preservation highlights the superior protective mechanism of IP, which aligns with the findings in 3.4.2 and further underscores its potential for maintaining the overall quality and texture of abalone over extended storage periods.

### 3.5 Correlation analysis

**Fig. 8:**
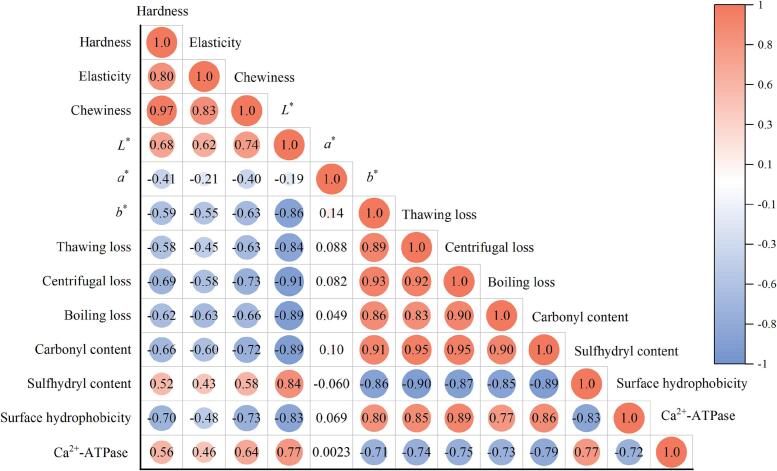
Correlation analysis between quality characteristics and protein oxidation of abalone. Red indicates a positive association, and blue indicates a negative correlation. The stronger the association and the deeper the hue. (For interpretation of the references to color in this figure legend, the reader is referred to the web version of this article.)

As shown in Fig. 8, Pearson coefficient was used to analyze the correlation between quality characteristics and protein oxidation. The texture (hardness, elasticity and chewiness) and color were positively correlated with sulfhydryl content and Ca^2+^-ATPase, while negatively correlated with surface hydrophobicity and carbonyl content. Conversely, the water loss (thawing loss, centrifugation loss and boiling loss) showed opposite trends. Notably, the L* value (*r* = −0.89, *p* < 0.01) and centrifugal loss (*r* = 0.95, *p* < 0.01) were significantly correlated with carbonyl content. It is speculated that the formation of carbonyl groups induces the oxidation of amino acid residues, protein structural modifications, which affect the reflection and absorption of light. Additionally, carbonyl-mediated cross-linking reduces water-binding capacity by decreasing hydrophilic groups and disrupting the protein matrix, explaining the strong link to WHC (Lin et al., 2022). The hardness (*r* = −0.70, *p* < 0.01) was significantly correlated with surface hydrophobicity, which due to the hydrophobic residue exposure, promotes protein-protein interactions, network formation and cross-linking density enhancing mechanical strength. Furthermore, the results of this study showed that there was no significant correlation between a* value and protein oxidation in frozen abalone.

## 4 Conclusions

In this study, the effects of different packaging methods—vacuum packaging, ice-coating, air-permeable polyvinylchloride, and non-packaging—on the oxidation characteristics, structural changes, and sensory quality of abalone muscle proteins during 24 weeks of frozen storage at −20 °C were systematically investigated. The key findings are summarized as follows:

(1) Frozen storage significantly induced the oxidation and degradation of proteins, which were manifested by the decomposition of high-molecular-weight proteins (such as MHC and paramyosin), the increase of carbonyl content (from 1.64 to 3.12–4.13 nmol/mg), the decrease of sulfhydryl content (20.48–29.94 %), the increase of surface hydrophobicity (19.50–40.24 %) and the decrease of Ca^2+^-ATPase activity (21.13–54.93 %). In addition, the secondary structure of the protein changed from α-helix and β-sheet to random coil, and the change of aromatic amino acid microenvironment in the tertiary structure further indicated the degradation of protein function. Among them, VP effectively alleviates oxidative stress and maximizes the conformational integrity of proteins by reducing oxygen exposure.

(2) Compared with other packaging, ice-coating significantly slowed down the color change (4.12–12.75 %) and texture deterioration (5.17–16.91 %) of fresh abalone caused by freezing, which are critical quality indicators influencing the price positioning of frozen abalone. During the 24 weeks frozen storage period, ice-coating packaging slowed down color changes among 8 ∼ 19 weeks and preserved the texture of fresh abalone among 12 ∼ 16 weeks in average.

(3) Texture and color exhibited positive correlations with sulfhydryl content and Ca^2+^-ATPase activity, while showing negative correlations with surface hydrophobicity and carbonyl content (*p* < 0.05). In contrast, water loss showed opposite trends. Notably, the L* value (*r* = −0.89, *p* < 0.01) and centrifugal loss (*r* = 0.95, *p* < 0.01) were significantly correlated with carbonyl content, while hardness (*r* = −0.70, *p* < 0.01) was significantly correlated with surface hydrophobicity. Thus, carbonyl content and surface hydrophobicity are the primary factors regulating color and texture quality.

(4) At the 8th week, the sulfhydryl content of each packaging group lost more than 77.08 % of the entire frozen storage period. Similarly, the quality characteristics WHC also showed the same trend. At the 8th week, the centrifugal loss reached more than 70.25 % of the total loss. Thus, 0 ∼ 8w is effective period in protein oxidation control considering oxidation markers and quality control by WHC trend.

This study provides practical theoretical support for production enterprises, distributors, and consumers alike. The −20 °C storage temperature offers valuable guidance for industrial applications, while emerging rapid-freezing technologies, particularly those focusing on moisture control, show significant potential for mitigating protein damage and improving the textural quality of frozen abalone. Furthermore, novel approaches to protein oxidation control are expected to enhance multiple quality attributes, including color and texture. Extending the shelf-life while maintaining premium quality through advanced preservation technologies represents a crucial research direction for maximizing the commercial value of marine products.

## CRediT authorship contribution statement

**Siyuan Zhu:** Writing – original draft, Software, Methodology, Investigation, Data curation, Conceptualization. **Chen Zhang:** Methodology, Investigation. **Yijun Liu:** Writing – review & editing, Resources, Methodology, Funding acquisition, Conceptualization. **Dan Jiang:** Validation, Supervision, Resources, Funding acquisition. **Qiancheng Zhao:** Supervision. **Xiqin Mao:** Supervision, Software. **Xia Hu:** Supervision. **Bohai Jiang:** Supervision, Software.

## Declaration of competing interest

The authors declare that they have no known competing financial interests or personal relationships that could have appeared to influence the work reported in this paper.

## Data Availability

Data will be made available on request.

## Appendix A Supplementary data

Comparison chart of actual abalone pictures at 0, 4, and 24 weeks during −20 ℃ storage under different packaging. Image 1
