# Supplementary material for: Effect of protein oxidation on the quality of abalone (Haliotis discus hannai) during frozen storage under different packaging conditions
Source: Food Chem X. 2025 Mar 10;27:102357. doi: 10.1016/j.fochx.2025.102357 (PMC11960662; doi:10.1016/j.fochx.2025.102357)
Supplement: Supplementary file 1 — Comparison chart of actual abalone pictures at 0, 4, and 24 weeks during −20 ℃ storage under different packaging. [file mmc1.docx]

**Supporting information for:**

**Fig. S1.** Comparison chart of actual abalone pictures at 0, 4, and 24 weeks during −20 ℃ storage under different packaging. Note: A-1: Fresh abalone; B-1 to B-4: Abalone at the 4th week during −20 ℃ storage under VP, IP, AP, and NP; C-1 to C-4: Abalone at the 24th week during −20 ℃ storage under VP, IP, AP, and NP.
